# Dealing with dissent from the medical ranks: Public health authorities and COVID-19 communication

**DOI:** 10.1177/09636625231204563

**Published:** 2023-11-16

**Authors:** Øyvind Ihlen, Anja Vranic

**Affiliations:** University of Oslo, Norway

**Keywords:** health communication, rhetoric of science and technology, science communication, science experts, scientific controversies

## Abstract

During a public health crisis like the COVID-19 pandemic, the public health authorities will typically be criticized for their efforts. When such criticism comes from the ranks of medical personnel, the challenge becomes more pronounced for the authorities, as it suggests a public negotiation of who has sufficient expertise to handle the pandemic. Hence, the authorities are faced with the challenge of defending their competence and advice, while at the same time adhering to a bureaucratic/scientific ethos that imposes communicative boundaries. This explorative study analyzes the response strategies used by the Norwegian public health authorities in this regard. A main finding is that the authorities shunned aggressive language and mostly relied on a strategy pointing to well-established values such as proportionality (between the measures and the gravitas of the epidemiological situation) and relevance (the measures should meet the challenge in question).

“We are simply not in safe hands. [The public health authorities] have not proven to be competent [to tackle the pandemic]” (Lien and Mogen, 2020). This was a statement from an MD specializing in infections, and it was printed in a large Norwegian newspaper during the early stage of COVID-19. The quote illustrates how there will be divergent opinions about the right course of action in a public health crisis such as this. The question of who has the relevant expertise to cope with the crisis becomes an issue, and the public health authorities are likely to be challenged. When the criticism comes from a source with relevant and recognized expertise, it poses a communicative challenge for public health authorities that seek to confirm their expertise status in the eyes of the public and political decision-makers. There is a lot at stake in this situation, since potential widespread doubt about their capability might damage trust and support for suggested measures (see also Jauho, 2016; Leidecker-Sandmann et al., 2022; Majid et al., 2020).

Who is an expert? Some research has explored general strategies to claim expertise and who the public considers to be experts (e.g. Coen et al., 2020; Jauho, 2016; König and Jucks, 2019; Suldovsky et al., 2019). Contrary to declarations about “the death of expertise” (Nichols, 2017), it has been asserted that those who want to criticize a particular scientific viewpoint have to make some kind of reference to competing scientific claims (Peters, 2021). In general, survey research indicates positive public attitudes toward experts (Dommett and Pearce, 2019). Importantly, an argument has also been made that such processes can be fruitful and that dominant expert views need to be exposed to criticism (Dommett and Pearce, 2019; Gesser-Edelsburg et al., 2021). Indeed, a public institution like a health authority must acknowledge the democratic value of dissent and debate, and cannot publicly stifle public accusations with aggressive responses (Kettle, 2008). At least it seems plausible that it would cause a stir if a public authority forcefully attacked the credentials of the critics, and sowed doubt about the motives or scholarly records, not to mention if the authorities used pejoratives in this regard. Pathos-free rhetoric, impartiality, and impersonality are longstanding bureaucratic values (Du Gay, 2005; Weber, 2016). This also means that public institutions and those complaining about their work or their decisions have different rhetorical opportunities (Ihlen & Thorbjørnsrud, 2014a, 2014b).

In science too, several similar values as those of the bureaucracy are seen as important (Constantinides, 2001). In general, it has been shown how aggressive language hurts trustworthiness in scientific debates (König and Jucks, 2019). Yet, beyond the advice against such language there seems to be little guidance to be found in the literature on the public understanding of science (PUS) on how to reaffirm authoritativeness and defend suggestions and policies on a scientific matter. This article will thus focus on the communicative boundaries constituted by a bureaucratic as well as a scientific ethos when attempting to meet criticism. Such boundaries, we suggest, imply that there are certain limits for what an actor can say and do without losing trustworthiness.

A rhetorical framework has been discussed in the PUS-context (Condit et al., 2012; Gross, 1994) and can be usefully applied to discuss how the perceived expertise of an institution forms part of its trustworthiness, which in turn can strengthen trust (e.g. McCroskey and Young, 1981; Mihelj et al., 2022). Studies have also shown how experts communicatively establish themselves as such, for instance, pointing to membership in networks of experts (Hartelius, 2011). Furthermore, rhetorical studies have detailed how individuals as well as organizations defend themselves against accusations of wrongdoing related to character or policy (Hearit, 2018; Ryan, 1982). The goal of such endeavors is to present a compelling counter-narrative of the actions that have been undertaken. Similarly, in crisis communication, response strategies have been researched and matched to the type of crisis and perceived responsibility (Coombs, 2012, 2022). These strands of research offer much valuable insight that can help address parts of the challenge described above, but neither of them alone offers a full answer to how a *public* institution can meet criticism of its *competence* (rather than being accused of a wrongdoing) leveled by *experts* (suggesting a certain validity of the criticism). As suggested above, there will be certain communicative boundaries in such situations. Thus, the research question pursued here is: *what response strategies are available for public health authorities confronted with expert criticism of their actions and recommendations?*

To answer the above research question, an explorative case study was conducted of how the Norwegian Institute of Public Health (NIPH) and the Norwegian Directorate of Health (NDH) answered criticism from health experts during the first 12 months of the COVID-19 pandemic. NDH coordinated the crisis management between the different sectors during the pandemic, while NIPH made recommendations based on their own research and collated scientific evidence. As will be explained in the section on methodology, four medical experts were particularly active in criticizing these authorities and argued for stronger measures to combat the pandemic.^1^ Combined, these four critics generated 249 news stories in the period during January 2020–December 2020 (the section on methodology provides further details). Despite the criticism, the level of trust in the public health authorities remained high throughout the pandemic (Directorate of Health, 2022; NOU, 2021) and higher than in the neighboring country of Sweden that chose a different route (e.g. Johansson and Vigsø, 2021). While this has been explained as a result of the perceived successful handling of the pandemic (e.g. a low fatality rate), communication is also said to have played an important part (NOU, 2022). One study highlighted factors such as “competent politicians, a high-trust society with a reliable and professional bureaucracy, a strong state, a good economic situation, a big welfare state, and low population density” (Christensen and Laegreid, 2020: 774). While strong “rally around the flag”-effects have been found in many countries (Van Aelst, 2022), a more modest effect is seen in Norway where trust in the public health authorities more or less remained high throughout the pandemic (OECD, 2022). This makes Norway a particularly apt case to study handling of criticism from the medical ranks (for more detailed descriptions of the Norwegian context, please see [Ihlen et al., 2022b; Johansson et al., 2023]).

Next, the theoretical approach is discussed more thoroughly. Second, details are provided about the methodology; third, the analysis is laid out; and finally, a discussion and conclusion are presented along with limitations of the study, as well as avenues for future research.

## 1. Theoretical framework

### Expertise rhetoric

Expertise is fundamentally rooted in communication (Suldovsky et al., 2019). Some research has also demonstrated the fluidity of the very concept through exploring the different discursive strategies people employ to position themselves as experts. Analyzing the comments sections of online newspapers in the United Kingdom and Germany, scholars found that readers relied on strategies of self-presentation (e.g. entitlement or use of expert language) and/or the construction of an argument as factual (e.g. providing sources, making appeals to common sense) (Coen et al., 2020).

Similarly, in a study cutting across four different spheres of public discourse—political, historical, medical, informational*—*recurring rhetorical patterns were in use when expertise was negotiated (Hartelius, 2011). Six discursive patterns typically constituted expertise: Expertise is signaled through (1) association of a wider network of experts and/or, (2) explication of specialized language and epistemologies—they “state what they know, how they know it, and how they practice or implement what they know” (Hartelius, 2011: 20). (3) Experts might also decide to rely on pedagogy and share insights in the process and the accompanying uncertainties, which ultimately may also lead to (4) the expert inviting the public along (or the opposite, arguing that the field is too complex and difficult to understand).

Another common feature of expertise rhetoric is that the expert constructs their expertise as (5) what is needed in the situation. And finally (6) expert rhetoric frequently claims relevance to everyday life. “The more relevant an expert seems to the public, the more powerful she will be” (Hartelius, 2011: 29).

Other studies have suggested that all these congruities should be used by those wishing to establish expertise, depending on the situation (Kjeldsen et al 2022a, 2022b). In expanding on the implications of a particular case, the latter study argues for use of invitational rhetoric that utilizes plain language related to everyday contexts. In addition, it points to the benefit of building alliances and strengthening networks, indicating the importance of the first discursive congruity.

Taken together, the expert position and the strategies to achieve this have an ultimate persuasive goal, namely that of being trusted. This is the *sine qua non* in the rhetorical process (Gross, 1994: 4). While rational arguments might be valid and important in this regard, they do not necessarily secure adherence to what is communicated; listeners choose to trust or not trust the communicator (Baumlin and Scisco, 2018). To this end, the expert must come across as trustworthy (Condit et al., 2012; Constantinides, 2001; Gross, 1994). In ancient rhetoric, the argument was that a speaker should *demonstrate practical wisdom, virtue*, and *good will* toward the audience (Aristotle, 2007). More recent research on processes of persuasion has produced similar categories of competence, character, and good will (McCroskey and Young, 1981; O’Keefe, 2016). A premise for this article is that when critics argue that the public health authorities are mistaken in their suggestions, this is a challenge or an outright attack on the competence of the authorities and their trustworthiness. Thus, it is not unlikely that they will attempt to reconfirm their expertise status by responding (Kjeldsen et al., 2022a, 2022b).

### Criticism and response

An analysis of the challenge to the competence of the public health authorities and their response might in part be inspired by looking at studies of the dual concept of *kategoria* and *apologia.* The former entails an accusation of a wrongdoing and the latter the subsequent speech of self-defense (Hearit, 2018; Ryan, 1982). Rather than being one-off incidents, the kategoria/apologia exchange should be perceived as a process. A refutation from the public health authorities might lead to more news stories or public debate (Vigsø, 2012).

The kategoria/apologia process and contest over expertise is played out publicly in the media arena. If the critics have some kind of credentials, they certainly strengthen the conflict and drama dimension that journalists tend to crave (Harcup and O’Neill, 2017). Research on previous pandemics has shown how dissident expert views typically appear later in the process, and that it is the public health authorities that drive the media coverage in the first phase (Vasterman and Ruigrok, 2013).

Scholars have typically separated between accusations concerning character (moral nature, motives, or reputation) and accusations concerning policy (Ware and Linkugel, 1973). Concerning the former, apologia strategies include “the allegations are false” (denial), “we all have a share in this” (bolstering), “this will be seen differently in the light of time” (differentiation), and “this belongs to a bigger picture” (transcendence) (Ware and Linkugel, 1973). When kategoria deals with policy, apologia strategies are said to fall in one of four categories: “I did not do it” (fact), “I did not do what is alleged” (definition), “I had laudable intentions” (quality), or “I appeal to another audience” (jurisdiction) (Ryan, 1982). More recent research has applied such perspectives in the context of organizations as well as developed further insights into the rhetorical operations of apologia particularly in crisis communication (Hearit, 2018). If an organization does respond in a crisis, the strategies are typically grouped in one of three clusters: deny, diminish, and deal (Coombs, 2012). The former includes responses stating that no crisis exists, while the second seeks to introduce elements that will have the public look more favorably on the organization, since the crisis is not that severe or the organization cannot be blamed. The third cluster includes acceptance of responsibility and attempts to rectify the situation. The case at hand—criticism of public health authorities—can hardly be said to constitute a crisis for the mentioned public institutions or to involve wrongdoing as such. Yet, there is inspiration to be found in the way that the response strategies can be located on a continuum with defensive responses on one side and accommodative ones on the other.

Importantly, studies indicate that response strategies cannot be chosen at will. First off, they must match the situation (e.g. the crisis type) (Coombs, 2022) and the actor type. As mentioned in the introduction, a public sector organization needs to adhere to its bureaucratic ethos, which in turn invites a subdued tone, reflecting the power discrepancy between the public institutions and individual critics (e.g. Du Gay, 2005). As such, this might go hand in hand with the cited finding that trustworthiness and credibility is hurt if aggressive language is used in scientific debates (König and Jucks, 2019).

### Public debate

A premise for the present study is that the public health authorities cannot quash criticism. From a societal and democratic viewpoint, public debate about measures to cope with a public health crisis has intrinsic value. In a crisis of this magnitude, it is more than ever a need to “test competing interpretations and challenge narrow perspectives” to avoid the type of reductionism that leads to a choice between the ultimate good and the ultimate bad (Ivie, 2002: 283). Indeed, taking a cue from political theory, the productive aspect of conflict can be embraced. Mouffe (2013), for instance, argues against liberalist or rationalist versions of democratic theory that sees rational debate as being able to forge a universal and inclusive consensus. There will always be division and power. When “the inescapable moment of decision” arrives, the limit of any rational consensus is demonstrated by remaining antagonism (Mouffe, 2013: 3). This line of thinking can be fruitfully transferred to science and public health issues, presenting a pluralistic imperative ensuring the value of deliberation. This seems particularly pertinent in the face of uncertainty presented in a crisis like the COVID-19 pandemic. The pandemic has been characterized by a high degree of ontological uncertainty and complexity. Thus, as stated in one study, “[c]ontroversy must be encouraged to prevent misconceptions” (Gesser-Edelsburg et al., 2021: 2553).

Yet, the media arena where the public debate takes place also introduces some constraints for both kategoria and apologia statements. For instance, journalists might simply use the frame of “expert controversy” (Peters, 2021), which is a dissatisfactory outcome for the public health authorities seeking a superior expert position. Furthermore, news tends to be brief, and there is absolutely no guarantee that the constitution of expertise in print or on air satisfies the expressed intent of the sources. At the same time, alarmist messages are considered attractive for journalists (Vasterman and Ruigrok, 2013). In a historical analysis of media coverage of epidemics, the author stated that the patterns in the coverage have remained consistent, issuing warnings about risks but also expressing hope of containment (Foss, 2020).

On the contrary, some research also indicates that the communication from experts has been different during the COVID-19 pandemic, for instance, with regard to the limits of scientific knowledge (Post et al., 2021), as well as transparency about uncertainty (Ihlen et al., 2022a; Paek and Hove, 2020). A recent German study also found that the media had predominately favored “reputable scientific expertise” to a greater extent than during previous pandemics (Leidecker-Sandmann et al., 2022: 1). If these findings are indeed correct, one might expect the mediated contest between experts to be somewhat reflective and contribute positively to the needed democratic debate about policy measures to fight a pandemic like COVID-19.

## 2. Methodology

Throughout the COVID-19 pandemic, the Norwegian news coverage was monitored closely to assess how the policy recommendations of the public health authorities were met. In this process, a number of critics from the medical profession were noted, of which four appeared as particularly active: (1) Bjørg Marit Andersen (BMA), Dr Med, Retired Professor of hygiene and infectious disease, Oslo University Hospital; (2) Jørn Klein (JK), PhD, Professor of microbiology and infectious disease, University of South-Eastern Norway; (3) Gunnar Hasle (GH), MD and Zoologist, PhD, Doctor at the Travel Clinic; and (4) Gunhild Alvik Nyborg (GAN), MD, PhD, researcher at the Department of Rheumatology, Oslo University Hospital. The central position of the first three was affirmed when they were addressed collectively in an op-ed piece signed by 35 infection control representatives from hospitals across Norway, professing support for NIPH (Akselsen et al., 2020). The latter—GAN—was included since she caused a national debate after her appearance on television in March 2020 (e.g. Hatlo, 2020).

While the first Norwegian cases of COVID-19 were reported on 26 February 2020 (Kalsnes and Skogerbø, 2021), already on 29 January 2020, BMA wrote an op-ed article, criticizing the general director of NDH for urging people to use their elbow hooks when coughing. Thus, using 1 January 2020, as the starting point, the coverage generated by the four critics was traced until 31 December 2020 2020 was the crucial year for this debate, particularly since the numbers showed how the pandemic was tackled rather successfully. Hence, the situation on the ground did not seem to call for stricter measures and thus the relevance of the criticism seemed to wane. Using the subscription-based, online newspaper archive Retriever, a research assistant traced the coverage, and a second research assistant repeated the search 1 year later. The search yielded 249 news stories.

The obtained texts were then coded by a research assistant specifically looking at which stories contained responses from the public health authorities (NIPH and NDH). The latter coding reduced the number of articles to 78 (31% of the 249 news stories). The responses were then analyzed inductively to identify what response strategies the public health authorities relied on. Apart from an “other” category, four clusters of responses could be discerned and distributed along a continuum where defensive and accommodative stances formed opposite poles: (1) denial: declaring that “the critics are wrong”; (2) epistemological network: implicitly saying the critics are wrong since the “authorities have the best available knowledge,” underscoring membership in epistemological networks of experts; (3) situational adaptation: the authorities have suggested proportional measures adapted to the situation; or (4) concessions: authorities indicated some agreement with the critics and that changes might be made.

A code book was then developed (available on request) based on this inductive analysis and then applied in a deductive coding process by a research assistant. While combinations of the strategies were found, the coders were asked to make a judgment of what strategy was most prominent, based on how much space was devoted to it. An intercoder-test was carried out by the second author, recoding 19 of the stories (24%). This resulted in a Kappa value of .647, which is considered substantial (O’Connor and Joffe, 2020).

## 3. Findings: Quantitative results

**Figure 1.**
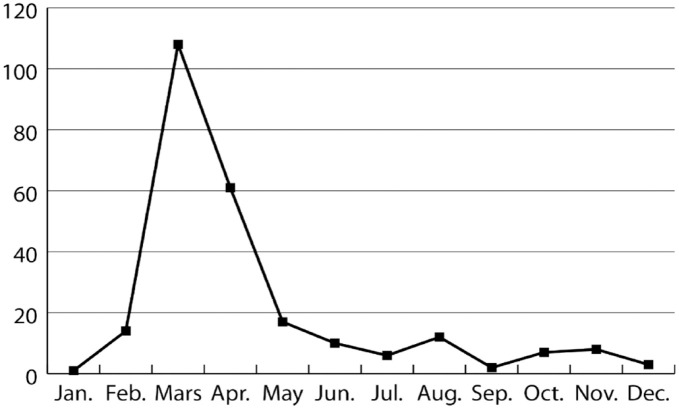
News stories quoting the critics (Jan 2020–Dec 2020, N = 249).

Figure 1 shows the distribution of the 249 news stories. There was a clear spike in March 2020, coinciding with the first outbreak and the ensuing lockdown in Norway. This then adds nuance to the findings from previous pandemics that dissident expert views appear at later stages and that the mentioned rally-around-the-flag effect blots criticism (Esaiasson et al., 2021; Van Aelst, 2022; Vasterman and Ruigrok, 2013). As shown, the general media coverage in Norway was not without dissenting voices in the first phase of the pandemic. Then again, an internal NDH document showed how NDH and COVID was mentioned 31,739 times from March to December 2020 and the similar figure for NIPH was 102,683. In other words, it could also be argued that the dissenting voices were largely drowned out by the sheer amount of coverage. Furthermore, apart from March and April 2020, the critics were not able to draw *substantial* attention. Yet, they were successful to the extent that infection control representatives wrote the mentioned op-ed article chastising the critics (Akselsen et al., 2020).

Table 1 shows the distribution between the four critics, of which two appear as particularly central—GAN and BMA—accounting for 43% and 33%, respectively (N = 249). BMA was active during the whole research period and also wrote 33 op-eds in this time span. GAN was primarily active in the early phase.

**Table 1.** Each critic’s proportion of the coverage (percentage).

	News stories
Bjørg Marit Andersen (BMA)	33
Jørn Klein (JK)	10
Gunnar Hasle (GH)	14
Gunhild Alvik Nyborg (GAN)	43
	100 (N = 249)

As mentioned, a total of 78 news stories contained responses from the health authorities. By far the most frequent response, as illustrated in Table 2, was the suggestion that the measures the authorities recommended were “proportional” to the challenges in the situation. The distribution across the other response categories was more or less even.

**Table 2.** Health authorities’ response strategies (percentage).

Denial	15
Epistemological network	14
Situational adaption	45
Concessions	14
“Other”	12
	100 (N = 78)

Ultimately, however, it must be remarked that the study is largely explorative in nature. The main goal is not quantification, but to generate ideas about what broader response strategies are available for public health authorities when faced with dissent from medical professionals. The rest of the analysis is based on a close reading of the responses in the present case. The quotes that are used in the analysis are translated by the first author.

## 4. Findings: Qualitative analysis

### Denial: “The critics are wrong”

As could be expected from the literature review (Du Gay, 2005; Kettle, 2008; König and Jucks, 2019), there is no condemnation of the critics in the material. For instance, on 28 March 2020, when one of the critics expresses fear that use of unskilled labor in the treatment of COVID-19 patients will increase infection rates, an NDH director simply says they trust that operating rules are followed and necessary training provided (Paust, 2020). This, we argue, is an example of a communicative boundary by the bureaucratic and scientific ethos.

The denial strategy is also evident when the public health authorities explicitly “disagree” with the criticism. One example is from 15 May 2020, when one of the critics argues that if it is okay to travel in Norway, Norwegians should also be allowed to travel in Demark, given the low infection rates there. The Acting Director of NDH says he disagrees:

The level of infection is relatively high in many places in the world, except for Iceland and Norway, and when you are traveling you are in more contact with people than otherwise. We end up in queues, travel by bus, train, plane etc., so this must be taken into account. (Lilleås, 2020)

Here then, the apologia takes the form of a counternarrative which also relies on examples from everyday life, a known strategy to strengthen an expertise position (Hartelius, 2011).

Probably the strongest counterreaction found in the material is the response to the accusation on 7 September 2020, that NIPH has presented the public with a “white lie” about face masks. Instead of pointing to a shortage of supplies, NIPH allegedly stated that face masks do not work. A Department Director of NIPH responds:

This is a gross claim, and of course completely wrong. [. . ..] The most important reason why we did not advise everyone to use a mask at that time [Spring 2020] was that we were unsure whether this would have a good effect on infection prevention. The knowledge base was fragile and fragmented. (Myrvang et al., 2020)

The flat-out rejection primarily relates to the accusation of lying, while the latter part of the statement points to the circumstance of uncertainty. As stated elsewhere, transparency about uncertainty can help strengthen trustworthiness under certain conditions (Ihlen et al., 2022a). The latter part of the response can thus also be called a differentiation strategy, seeking to separate *the recommendation given at the time* with *the recommendation that would be given in light of new circumstances and evidence* (Ware and Linkugel, 1973).

### Epistemological network: “We have the best available knowledge”

Another strategy—slightly more subtle refutation than outright denial—is when the authorities state that their advice is based on information from an international expert network. On 7 February 2020, one of the critics asks the authorities to act preemptively since the situation is so uncertain. A director at NIPH agrees to the description of uncertainty, but also argues that “NIPH bases its advice on information from WHO and ECDC (European Centre of Disease Control)” (NTB, 2020).

In other words, NIPH is part of a larger network of international experts and this expertise has not recommended lockdowns or similar measures. Again, this is a form of discursive congruity recognized in expertise rhetoric (Hartelius, 2011). It is also a bet on the ethos—or more precisely the perceived competence—of these other institutions. Debating face masks, on 16 April 2020, a section head at NIPH says the ECDC reports how “use of face masks can create a false sense of security and make people less likely to follow advice from the authorities about social distancing” (Lode et al., 2020).

In some instances (e.g. Lode et al., 2020), the reference to a wider network of experts is also combined with what turned out to be the main line of response—the insistence on the need for situational adaption. On 7 March 2020, for instance, a senior NIPH doctor states as follows:

We are in continuous contact with the WHO and follow the recommendations of the European Centre of Disease Control. We also have close and good contact with the other Nordic countries to coordinate along the way. (Wernersen, 2020)

Despite how some other research has pointed to the importance of the strategy of pointing to an epistemological network (Kjeldsen et al., 2022), it is not frequently found in the empirical material gathered for the present study. There are several hypotheses that might explain why the network strategy was not used more frequently, for instance, that the short news stories did not have space enough for the argument. Another possible explanation would be that potential mistrust in the international institutions made the strategy less attractive. A third possibility is that the strategy was shunned, since it may come across as an attempt to resolve the institution of responsibility or that it indicates a lack of agency. Indeed, a later accusation from one of the critics would be that the Norwegian authorities were just trailing “reluctantly behind the rest of Europe” (Hella, 2020).

### Situational adaption: “The suggested measures are proportional”

During the buildup of the pandemic, the critics’ call for introduction of stricter border controls and travel bans is typically met with arguments for a “knowledge-based” approach. On 9 February 2020, for instance, an NIPH director responds to the call for screening of passengers arriving at Norwegian airports:

Our role is to give advice based on the knowledge we have. This means that we are open to the fact that advice can be changed along the way when we gain new knowledge. We believe [. . ..] we take account of the most likely infection, given the knowledge we currently have. (Lien, 2020)

As such, this response illustrates an approach to risk that favors a scientific strategy over the precautionary one suggested by much research on risk governance (Aven and Renn, 2020; Stirling, 2007). Importantly, the knowledge-based approach invites proportionality between the risk and measures. On 9 March 2020, an NIPH director says: “We are constantly concerned that the measures must be highly knowledge-based, and they must be proportionate to the danger posed by the epidemic” (Mo, 2020). The ability to match the best available knowledge with what is needed in a current situation can also be construed as a form of expertise rhetoric (Hartelius, 2011).

On 12 March 2020, a partial lockdown is introduced in Norway. While the critics still call for a stricter version, the response from NIPH remains the same: “We have been keen to have measures that are commensurate with the risk we are aware of” (Hatlo, 2020). Later the same month, when the testing capacity is criticized, proportionality is also heralded by an NIPH director:

Right now, it’s about testing vulnerable groups, people who are already hospitalized, people who are being admitted to hospital and healthcare personnel. Continuous assessments are made, but we believe that the test regime we now have is reasonable in relation to the capacity, a capacity that is at the top of the world. (Fossheim and Braaten, 2020)

The same argument is made 5 April 2020, discussing whether face masks should be recommended. The acting director of NDH states:

Given the infection situation in Norway today, both the [NIPH] and the Directorate of Health consider that the general use of masks in public spaces has little merit and is unfortunate because it deprives the health services of equipment that is in great need. (Fjeld and Mogen, 2020)

If accepting the premise of the need for a knowledge-based policy and the topos of proportionality, this then forms a compelling counternarrative to the criticism and calls for stricter measures (Hearit, 2018). At the same time, this strategy also signals flexibility. If the situation changes—new knowledge is acquired—the advice, and the policy, might change as well. The position thus functions as a hallmark of rationality and can also be linked to the fourth strategy of making concessions to the critics.

### Concessions: “Changes will be made”

The fourth response type found in the material relates to how the public health authorities signal agreement with the critics. One example is, for instance, when the lack of protection equipment is criticized and the NDH agrees that this has not been high enough on the agenda in the pre-COVID days (Næss, 2020). Another example is from 11 March 2020, when airplanes from Italy are allowed to land in Norway despite the widely covered catastrophic COVID-19 situation in certain Italian regions. The authorities signal that changes will be made. The General Director of NDH says, “We want [the passengers] to be stopped before they board the plane. It may take time before we have [regulations] in place. We inform the countries, which inform their citizens that if they travel [to Norway], they will be quarantined” (Rangnes, 2020).

Another example that is also related to the third response strategy (situational adaption) is when one of the critics questions the policy on how long people should be in quarantine before they no longer infect others. An NIPH representative says “The margin one should have after the symptoms are gone is being discussed [. . .]. We are waiting for several new research reports. Our advice will be updated continuously upon receiving research” (Andreassen, 2020).

Similarly, in August 2020, when a critic again calls for mandatory face mask use, an NIPH physician talks about a new assignment from the Ministry of Health and Care:

As part of the assignment, [NIPH] is now making a new assessment of whether face masks are a measure we should recommend in certain contexts. [. . .] It may [. . .] be appropriate to recommend the use of a mask as a supplement to the basic infection control advice, also to prevent the increasing spread of infection. Masks cannot replace advice such as distance between people but will to some extent be able to compensate for less distance. (Relling, 2020)

As already stated, the willingness to change can be looked upon favorably and strengthen trustworthiness as it demonstrates integrity (Kjeldsen et al., 2022a; Kjeldsen et al., 2022b). It is also in line with the suggestion that science should be communicated as something that is evolving (Hodson et al., 2023). Hence, this signals that a science-based policy should not be expected to remain the same throughout a highly uncertain event like a pandemic.

## 5. Discussion and conclusion

The public understanding of science and the right course of action in a pandemic like COVID-19 is necessarily influenced by which experts are trusted. This explorative study does not engage with the substance of the criticism nor the rhetorical strategies of the critics’ attempts to claim expertise (e.g. Coen et al., 2020) in this particular case. The focus is instead on the communicative choices available for an organizational entity that seeks to maintain an expert position and defend a practice, while at the same time operating within the communicative boundaries constituted by a bureaucratic as well as a scientific ethos (Du Gay, 2005; Kettle, 2008; König and Jucks, 2019; Weber, 2016). In order to preserve the latter, the public health authorities did not aggressively pursue the critics or brush aside their arguments. The value of deliberation is important to uphold in a social democratic context like Norway.

The study has argued that the responses public authorities might give to criticism of their competence can be placed on a continuum from the defensive to the accommodative, much like response strategies in crisis situations in general (Coombs, 2012; Hearit, 2018). Four archetypes are suggested in this regard—denial, emphasis on epistemological superiority through an epistemological network, situational adaptation and proportionality, and concessions. The strategy pointing to situational aspects was the preferred one in this case. This is a strategy whereby the authorities stand their ground, arguing from a well-established value (proportionality), while not necessarily engaging directly to refute the criticism. In other words, it provides the organization with flexibility, which might also help explain why it was favored. It also shuns the type of aggressive language that is detrimental to trustworthiness (König and Jucks, 2019) and strikes a balance between affirming a position and signaling willingness to adapt should the situation change. It is also a strategy recognized in the writings on expertise rhetoric, pointing to *relevance* for a situation (Hartelius, 2011). Taken together, it is not surprising that this strategy dominates in the material.

We have emphasized the communicative constraints that exist for public institutions operating in the realm of science. The study thus falls in line with how scholars have called for judgment, rather than mere description of the rhetoric in use (Condit et al., 2012; Gross, 1994). The mentioned welcoming of agnostic, rather than antagonistic, debate would invite dissenting voices, particularly from those with medial expertise. The level of ontological uncertainty is intimately tied to the epistemic uncertainties that also need to be addressed by communicators (Frewer et al., 2002; Paek and Hove, 2020). In such instances, others have urged that contexts of disagreement should be highlighted, but that there should “be at least some convergence of opinion before scientific views are communicated broadly to the general public” (Paek and Hove, 2020: 1731). Perhaps paradoxically, what could be perceived as an undermining of the expert position of a public health authority might function to strengthen this position by increasing trustworthiness (Kjeldsen et al., 2022a; 2022b).

With the chosen research design, there are certain limitations. First, it is obviously not possible to confirm causality between the rhetorical strategies and the mentioned high levels of trust in the public health authorities (Christensen and Laegreid, 2020; Directorate of Health, 2022; NOU, 2021). What can be said, however, is that the critics apparently did not influence the trust negatively. One hypothesis could be that the response strategies of the public health authorities had a positive influence in this regard. Further research could be carried out to identify the effect of different response strategies through, for instance, communication experiments or focus group research. Or, in keeping with what are typical contributions from rhetorical studies (Condit et al., 2012; Gross, 1994), more case studies could be accumulated to test the presented conclusions. Given that the context of the present study is a country with a relatively consensual political culture (Knudsen, 2020), it might also be worth testing the assumption about negative effects stemming from aggressive responses. Again, the mentioned survey research focusing on trust in public health authorities during the pandemic at least indicated that refraining from such responses did not hurt trust.

## Notes

1. The opposite view from medical experts was less prominent in public. For instance, a search in the database Retriever yielded only 67 news stories about the Great Barrington Declaration against lockdown measures, which was signed by several Norwegian MDs.
